# Wheat and Cereal Rye Inter-Row Living Mulches Interfere with Early Season Weeds in Soybean

**DOI:** 10.3390/plants10112276

**Published:** 2021-10-24

**Authors:** Charles M. Geddes, Robert H. Gulden

**Affiliations:** Department of Plant Science, University of Manitoba, 222 Agriculture Building, 66 Dafoe Road, Winnipeg, MB R3T 2N2, Canada; Charles.Geddes@agr.gc.ca

**Keywords:** allelopathy, cover crop, integrated weed management, inter-row mulch, living mulch, Northern Great Plains, plant competition, plant interference, seed return, weed suppression

## Abstract

Rapid growth of cool-season weeds in the spring exacerbates weed interference during early soybean (*Glycine max* (L.) Merr.) establishment in northern climates. This study tested the utility of spring-seeded inter-row living mulches in soybean for early season weed suppression using volunteer canola (*Brassica napus* L.) as a representative model weed species. The effects of the presence or absence of spring wheat (*Triticum aestivum* L.) or winter cereal rye (*Secale cereale* L.) living mulches (mulch type) that had been seeded simultaneously with soybean grown using 38 or 76 cm row spacing (spatial arrangement) and the presence or absence of herbicides used for mid-season mulch termination (herbicide regime) were evaluated in three environments in Manitoba, Canada, in 2013 and 2014. Soybean yield was similar in the presence and absence of the living mulches. In the environment that received the lowest precipitation (Carman 2013), the mulches terminated with post-emergence glyphosate resulted in a 55% greater soybean yield compared to the mulches that remained live throughout the growing season. Inter-row mulches that had been living or terminated mid-season reduced volunteer canola seed production by about one-third (up to 9000 seeds m^−2^). This study demonstrates the utility of wheat or cereal rye inter-row living mulches for enhanced interference with weeds during early soybean establishment.

## 1. Introduction

Soybean (*Glycine max* (L.) Merr.) and canola (*Brassica napus* L.) are two of the three most grown crops based on seeded areas in Manitoba, Canada, and common herbicide resistance traits in these crops can hinder weed control efforts [1,2,3]. Canola can become a problematic volunteer weed since seed lost during crop harvest [4] can enter secondary dormancy, resulting in a moderately persistent seedbank that persists throughout short crop rotations [5]. Proactive management, including the minimization of harvest losses [4,6] and increased seedbank decline with timely soil disturbance [7], can reduce volunteer canola population densities. However, uncontrolled volunteers in subsequent crops can return seed to the soil seedbank [8], resulting in population persistence and the emergence of this weed several years after canola production [9]. Thus, limiting volunteer canola seed production and return to the soil seedbank is imperative to mitigating the establishment of this weed in soybean production systems.

Volunteer canola is a cool-season weed that exhibits rapid growth and development in the early spring, providing it with a competitive advantage over soybean in the Northern Great Plains region of North America. Soybean row spacing, seeding density, and inter-row tillage did not reduce volunteer canola seed production when these tools were implemented alone [1]. Limiting the supply of soil nitrogen that was available for uptake by weeds was the only weed management tool in soybean that reduced volunteer canola seed production. Geddes and Gulden [1] attributed the limited response of volunteer canola to this suite of in-crop non-chemical weed management tools to the rapid growth and development of volunteer canola relative to soybean, particularly in the early growing season (early season). Thus, practices that target early season weed interference may aid in the management of volunteer canola and other cool-season weed species when soybean are grown in short-season environments.

Cover crops have multiple benefits in agroecosystems [10,11]. Recent studies have postulated the utility of cover crops for interference with herbicide-resistant (HR) weeds in HR cotton (*Gossypium hirsutum* L.) [12,13,14]. One such strategy could be the utilization of spring-seeded living mulches [10,15,16]. Living mulches are planted prior to or with a cash crop and are maintained as living soil cover throughout the growing season [10,15,16]. In these systems, living mulches may be tailored to reduce detrimental impacts on cash crop yield and facilitate weed suppression at the same time.

Plant interference comprises both resource-limiting (direct) and non-resource-limiting (indirect) competition [17]. Living mulches interfere with weeds directly via resource consumption [16] as well as indirectly through root exudates [18], volatile organic compounds [19], tissue leachate [20], or the microbial transformation of plant-derived compounds [21] (i.e., allelopathy). Aqueous extracts of rye (*Secale cereale* L.) and wheat (*Triticum aestivum* L.) tissues chemically inhibit canola radicle elongation in soil, and the vegetative tissues of these species have greater allelopathic potential than reproductive tissues [22]. Simultaneously seeding a winter cereal with a cash crop in the spring prevents the reproductive development of the winter cereal during that growing season [23] and may facilitate greater allelopathic activity during this timeframe.

In cover crop-based systems, cereal rye is often paired with soybean. In these systems, cereal rye is commonly established in the autumn and is terminated physically or chemically during anthesis the following spring [24,25]. Seeding rye living mulch into or with the cash crop can also facilitate interference with weeds [23,26,27,28]. Rye living mulch, however, can use soil moisture, which may have a detrimental effect on crop yield under moisture limiting conditions [23,29,30]. Mid-season mulch termination during soybean canopy development may be necessary to minimize mulch-induced yield loss and to maximize weed suppression early in the growing season [15,31].

Several factors influence interference from inter-seeded living mulches, including planting dates [27], plant densities [23], plant spatial arrangement, resource niches, and soil fertility [16]. For example, soybean yield loss was reduced with delayed rye inter-seeding [27]. However, if the early season suppression of cool-season weeds is sought in warmer-season crops—such as in volunteer canola in soybean—the simultaneous seeding of cover and cash crops could help balance early season interference with optimal crop yield.

The northern frontier of North American soybean production is located between 49° N and 51° N [1]. The limited duration of frost-free days in this region provides a narrow window for soybean production. Cool-season weed species are abundant in this region [32]. Many of these weed species grow rapidly in the early season. In other regions, rye living mulch has been effective for weed interference, but the impact of rye living mulch on soybean yield is dependent on the environment [23,29]. Spring wheat is a cool-season crop that can be competitive in the early season [33]. Spring-seeded wheat or cereal rye between soybean rows may provide early season interference with weeds in this short-season environment.

This study was designed to determine whether spring-seeded wheat and cereal rye inter-row living mulches could interfere with cool-season weeds in soybean without reducing soybean yield. The objectives of this study were to evaluate the effects of the (a) presence or absence of spring-seeded spring wheat or winter cereal rye living mulches growing between (b) 38 or 76 cm soybean rows and (c) the presence or absence of mid-season mulch termination on the management of volunteer canola in soybean.

## 2. Materials and Methods

### 2.1. Experimental Sites

Spring-seeded wheat and cereal rye inter-row living mulches in soybean were evaluated in three environments in Manitoba, Canada, based on management of volunteer canola. Volunteer canola was used as a model cool-season weed species that was representative of weed species that dominate weed communities in this region. Field research locations included the Ian N Morrison Research Station near Carman, Manitoba, in 2013 (49.48° N, 98.04° W) and 2014 (49.49° N, 98.04° W) and the Westman Agricultural Diversification Organization near Melita, Manitoba, in 2014 (49.41° N, 101.02° W). The previous crop at each location was wheat and field preparation included autumn tillage via tandem disc followed by spring cultivation. The soil characteristics for each environment were described in-detail by Geddes and Gulden [1]. In brief, the top 60 cm of soil ranged in NO_3_-N among environments from 11 to 37 kg N ha^−1^, and the 0–15 cm soil layer ranged in organic matter from 2.5% to 2.9% and in pH from 5.8 to 7.5. Soil textures were classified as clay loam (Carman 2013), sandy clay loam (Carman 2014), and loam (Melita 2014).

### 2.2. Experimental Design and Treatment Structure

The field experiment followed a split-block randomized complete block design with four experimental replications (blocks) per environment. The main plots (2.5 by 8 m) consisted of a two-way factorial of mulch type (unseeded vs. spring wheat vs. winter cereal rye) and spatial arrangement (38 vs. 76 cm soybean row spacing), while sub-plots (2.5 by 4 m) resulted from the application of either glyphosate or clodinafop assigned randomly to the front- or back-half of each block.

Soybean “DKL 23-10 RY” (seed treated with Acceleron^®^; Monsanto Canada Inc., Winnipeg, MB, Canada) was planted using either 38 or 76 cm row spacing, resulting in either one row or three rows of living mulch (19 cm mulch row spacing) between the soybean rows, respectively (hereafter referred to as “spatial arrangement”). The inter-row living mulches (hereafter referred to as “mulch type”) were seeded simultaneously with soybean and included either spring wheat “Kane” (71 kg ha^−1^ within-row seeding density), winter cereal rye “Hazlet” (78 kg ha^−1^ within-row seeding density), or an unseeded mulch control. Soybean was seeded at a depth of 2 cm and at a target density of 400,000 target plants ha^−1^ (455,000 seeds ha^−1^). TagTeam^®^ MultiAction^®^ granular soybean inoculant (Monsanto, St. Louis, MO, USA) was added in each soybean row at the recommended rate for each soybean row spacing (3.2 kg ha^−1^ in 38 cm and 1.6 kg ha^−1^ in 76 cm soybean rows). Prior to seeding the soybean, canola “DKL 73-45 RR” (seed treated with Acceleron^®^; Monsanto Canada Inc., Winnipeg, MB, Canada) was seeded at 1 cm depth (19 cm row spacing) along each experimental block (perpendicular to the soybean rows) at a density of 80 seeds m^−2^. A consistent density of volunteer canola was seeded in the experiment to mitigate the heterogeneous interference that can manifest from patchiness of weed establishment from the ambient seedbank. At soybean stage BBCH 13, glyphosate (Roundup WeatherMax^®^, 900 g a.e. ha^−1^, Monsanto Canada Inc., Winnipeg, MB, Canada) was applied for broad-spectrum post-emergence weed management in one half of each block and clodinafop (Horizon^®^ 240EC, 54 g a.i. ha^−1^, Score^®^ Adjuvant, 0.80 L ha^−1^, Syngenta Canada Inc., Guelph, ON, Canada) was applied for selective grass weed management in the other half of each block. In essence, glyphosate was used to terminate wheat and rye living mulches at stage BBCH 21, while clodinafop was used for the selective management of grass weeds while allowing the mulches that tolerate clodinafop to remain alive (hereafter referred to as “terminated” and “living” mulches, respectively). All herbicides were applied at 276 kPa using TeeJet^®^ AIXR 110015 nozzles (TeeJet^®^ Technologies, Wheaton, IL, USA) and with 100 L ha^−1^ water carrier.

### 2.3. Data Collection

#### 2.3.1. Soybean

The soybean response variables included seedling density, aboveground biomass, and grain yield. Soybean seedling density was determined by counting all seedlings in 1 m of two adjacent soybean rows at stage BBCH 10–12. Soybean biomass was determined at stage BBCH 77 by collecting all aboveground soybean tissue from one 50 by 50 cm quadrat with one side aligned with a soybean row. The biomass samples were dried at 60 °C until equilibrium, and dry weights were determined. At soybean maturity, each experimental unit was harvested using a Kincaid 8-XP plot combine (Kincaid Equipment Manufacturing, Haven, KS, USA), and harvest samples were air-dried and cleaned using a clipper M2BC seed cleaner (Blount/Ferrell-Ross, Bluffton, IN, USA). Soybean seed was separated from the wheat and volunteer canola using hand sieves (4.7 mm and 2.7 mm 136 round hole, Can-Seed Equipment Ltd., Winnipeg, MB, Canada). The soybean yield was adjusted to 13.0% moisture.

#### 2.3.2. Inter-Row Mulches

The inter-row mulch response variables included seedling density, plant height, aboveground biomass, and grain yield. Mulch data collection followed the methods used for the soybean response variables. In brief, the living mulch seedling density was determined at stage BBCH 13–14, and the mulch aboveground biomass was collected from the same sample area as the soybean biomass. Living mulch plant height was determined at 15, 30, 45, and 60 days after soybean emergence by measuring the height of three randomly selected plants per subplot from the soil surface to the distal end of the fully extended shoot tissue. In the spring wheat living mulch treatments only, the wheat entered reproductive development, producing an intercrop with measurable grain yield. The wheat grain yield was collected with soybean at harvest and was separated from the soybean seed using the hand sieves described above. The wheat yield was adjusted to 13.5% moisture.

#### 2.3.3. Volunteer Canola

The volunteer canola response variables included seedling density, plant survival to maturity, aboveground biomass, plant fecundity, seed production (per unit area), and the amount of seed returned to the soil seedbank (seed return). Volunteer canola seedling density was determined at canola stage BBCH 12–13 by counting all volunteer canola seedlings within two 50 by 50 cm quadrats with one side aligned with a soybean row. Volunteer canola aboveground biomass at canola stage BBCH 82 was determined following the methods described for soybean. Late-season canola densities were determined by counting the number of canola plants included in the biomass sample. The canola biomass samples were hand-threshed, and the seed was cleaned using a hand sieve (2.7 mm round hole, Can-Seed Equipment Ltd., Winnipeg, MB, Canada) and seed blower (Agriculex, Model CB-1, Guelph, ON, Canada). The total canola seed produced per unit area (g seed m^−2^) and individual seed weights (g thousand seeds^−1^) were determined. These total and individual seed weights were used to determine the number of volunteer canola seeds produced per unit area (thousand seeds m^−2^). Volunteer canola plant fecundity (no. seeds plant^−1^) was determined by dividing the number of seeds produced per unit area (no. seeds m^−2^) by late-season canola plant densities (no. plants m^−2^). The percentage of emerged volunteer canola seedlings that survived to maturity (%) was determined by dividing the late-season plant density by seedling density. Volunteer canola seed collected in the soybean yield sample (i.e., dockage) was separated from the soybean seed using the hand sieves described above. The individual seed weight (g thousand seeds^−1^) of the volunteer canola dockage was used to determine the number of volunteer canola seeds collected per unit area (no. seeds m^−2^) during combine harvest. Canola seed return was determined as a percentage of the total number of volunteer canola seeds produced (collected at biomass sampling) using Equation (1).

Seed return (%) = [(no. seeds m^−2^ at biomass − no. seeds m^−2^ in dockage)/no. seeds m^−2^ at biomass] × 100 (1)

### 2.4. Statistical Analysis 

The MIXED procedure in SAS 9.4 (SAS Institute, Inc., Cary, NC, USA) was used to analyze all data. The statistical model described a four-way factorial split-block RCBD. The four factors included two spatial arrangements (38 or 76 cm soybean row spacing), three mulch types (spring wheat, winter cereal rye, or unseeded (no mulch)), two herbicide regimes (glyphosate or clodinafop), and three environments (Carman 2013, Carman 2014, and Melita 2014). For each response variable, the Shapiro–Wilk test was used to assess the assumption of normality and visual inspection of residual vs. predicted values was used to examine homogeneity of variance [34]. Lund’s test was used to remove extreme outliers [35], and the square root-transformation was used when necessary to meet the assumptions of normality or homoscedasticity. The fixed effects for the analysis of all of the soybean and volunteer canola response variables included the main and interaction effects of spatial arrangement, mulch type, herbicide regime, and environment. The random effects included experimental block nested within the environment, the interaction effect of spatial arrangement, mulch type, and experimental block nested within environment, and the interaction effect of herbicide regime and experimental block nested within environment. The analyses of mulch seedling density and aboveground biomass focused on the subset of treatments containing living mulch only. The model for the analyses of mulch density and biomass used the main and interaction effects of spatial arrangement, response species (wheat or rye), and environment as fixed effects, while experimental block nested within environment was considered a random effect. The analysis of wheat intercrop yield focused on the experimental units containing living wheat only. The statistical model for wheat yield was reduced to include the main and interaction effects of spatial arrangement and environment as fixed effects and experimental block nested within environment as a random effect. Within each environment, living mulch plant heights were analyzed using a repeated measures RCBD [34]. The fixed effects included spatial arrangement, response species (wheat or rye), and measurement date, while experimental block was considered a random effect. In each analysis, measurements of the individual experimental units were repeated in time, and the toeplitz covariance structure was fit based on minimization of the Akaike information criterion (AIC) [34].

A hierarchical approach was used to trim the number of parameters in the models to achieve model parsimony based on the minimization of the AIC and the likelihood ratio test [36]. In addition to data transformation, the repeated within-group covariance structure of the residuals was selected based on the minimization of the AIC [37] to adjust for homoscedasticity. Post hoc mean comparisons were conducted using Tukey’s HSD (α = 0.05) [37], and mean letter separations were generated using the pdmix800 macro [38].

## 3. Results

### 3.1. Soybean 

**Figure 1:**
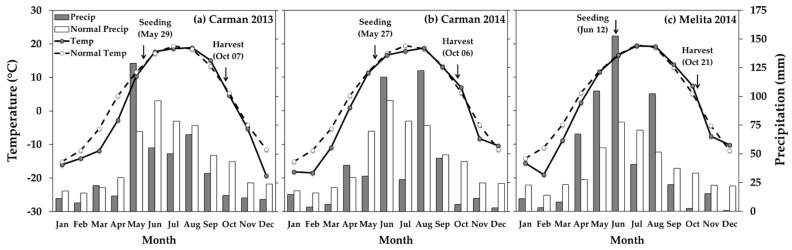
Mean air temperature (Temp; °C) and total precipitation (Precip; mm) for each month of the experiment and the regional climatic normal (1980–2010) air temperature (Normal Temp) and total precipitation (Normal Precip) for each month in (**a**) Carman 2013, (**b**) Carman 2014, and (**c**) Melita 2014. Seeding and harvest dates are indicated for each environment. Adapted from Environment Canada [39].

The presence of wheat or cereal rye inter-row mulches did not reduce soybean productivity under volunteer canola interference. Soybean yield remained the same in the presence or absence of the inter-row mulches, except when growing season precipitation was below normal. Under reduced precipitation, mid-season mulch termination was required to maintain soybean yield (Table 1; Figure 1). Carman 2013 was the driest growing season, with below average precipitation from June through October (220 mm precipitation in Carman 2013 vs. 319 mm in Carman 2014 and 321 mm in Melita 2014) (Figure 1). Increased water use by the living mulches during this timeframe likely contributed to the roughly 30% reduction in soybean yield compared to the mulches that were terminated mid-season (Table 1). Over double the mulch density at Carman 2013 (136 plants m^−2^ compared with an average of 52 mulch plants m^−2^ in all other environments) likely compounded the impacts of the living mulches on soybean yield under moisture deficit (Table S1). Inter-row mulches, live or terminated, did not impact soybean yield in the other two environments where growing season precipitation more-closely reflected that of the 30-yr climatic normal for this region (Table 1; Figure 1). Similar to yield estimates, soybean biomass was also about one third lower in the living vs. terminated mulch treatments at Carman 2013; however, the main effect of herbicide regime in this environment suggests that these differences in soybean biomass were also observed in the mulch-free control treatments (Table 2). Together, these data suggest that wheat or cereal rye inter-row living mulches can be effectively maintained in soybean production in the Northern Great Plains region with minimal risk to soybean yield. Mid-season mulch termination with post-emergence herbicide application can be used to mitigate the detrimental effects of the living mulch on soybean yield in years with below normal precipitation.

**Figure 2:**
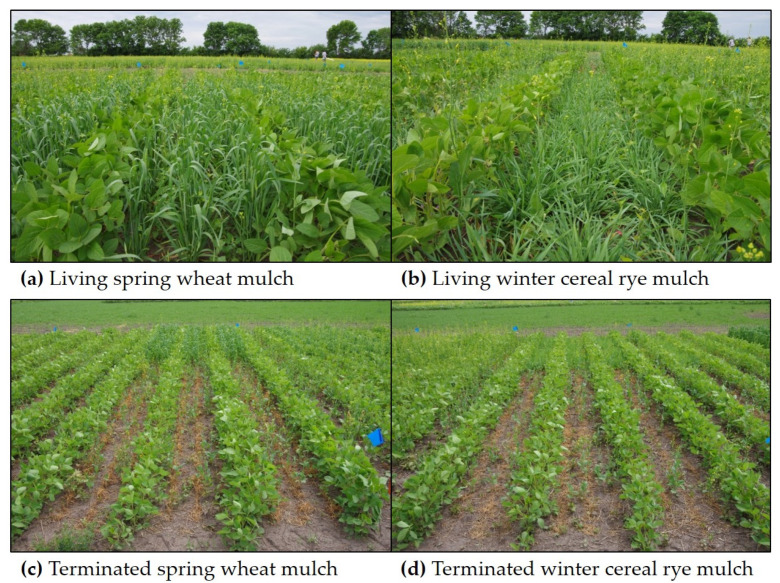
Spring-seeded (**a**) living wheat, (**b**) living cereal rye, (**c**) terminated wheat, and (**d**) terminated cereal rye inter-row mulches between soybean rows spaced 76 cm apart.

The soybean tended to yield greater and result in greater biomass when grown in alternating rows with the wheat or rye mulches (38 cm soybean row spatial arrangement) compared to growing three rows of mulch between each soybean row (76 cm soybean row spatial arrangement; Table 1; Figure 2). At Carman 2014 only, the 38 cm spatial arrangement resulted in a 55% yield increase over the 76 cm spatial arrangement (Table 1). This was likely due to greater soybean density when grown using the 38 cm (38 plants m^−2^) compared to the 76 cm spatial arrangement (20 plants m^−2^) in Carman 2014 only (Table S2); however, this trend was not significant in the wheat inter-row mulch treatments. In contrast, the main effect of spatial arrangement in Carman 2014 suggests that the 38 cm spatial arrangement resulted in greater soybean yield in both the presence or absence of the inter-row mulches (Table 1). The 38 cm spatial arrangement resulted in 22% greater soybean biomass among mulch types, herbicide regimes, and environments (Table 2). These results suggest that soybean may have a subtle yield advantage when grown in alternating rows with living mulch compared to three mulch rows between each soybean row.

### 3.2. Volunteer Canola

**Figure 3:**
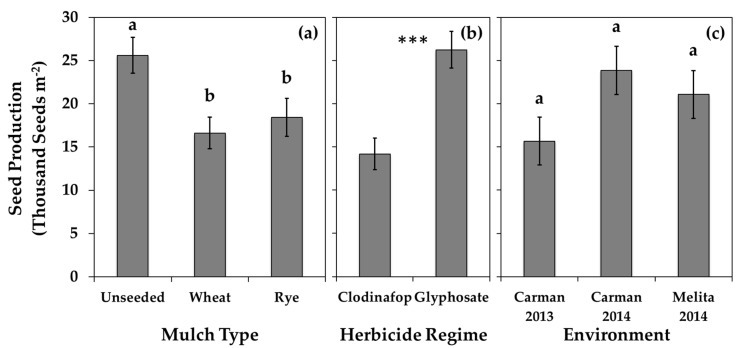
Volunteer canola seed production per unit area in soybean in (**a**) the presence or absence of spring-seeded wheat or rye inter-row mulches (mulch type) in a combined environment analysis, (**b**) two different herbicide regimes in a combined environment analysis, and (**c**) at each environment individually. Error bars indicate ± SEM. Within sub-figures where multiple comparisons are made, different letters indicate significant differences based on Tukey’s HSD (α = 0.05). Within sub-figures where a single mean comparison is made, *** indicates a significant F-test effect at *p* < 0.001.

**Figure 4:**
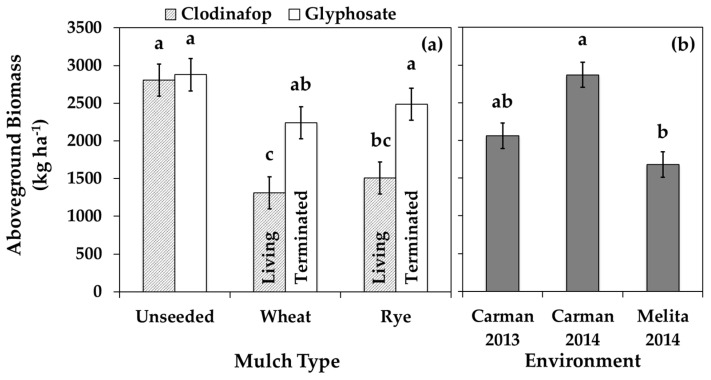
Volunteer canola aboveground biomass in soybean in (**a**) the presence or absence of spring-seeded wheat or rye inter-row mulches (mulch type) with (glyphosate) or without (clodinafop) mid-season mulch termination using two different herbicide regimes in a combined environment analysis and (**b**) each environment individually. Error bars indicate ± SEM. Within sub-figures, different letters indicate significant differences based on Tukey’s HSD (α = 0.05).

The spring-seeded inter-row mulches in soybean interfered with volunteer canola early in the growing season and reduced volunteer canola seed production consistently among the environments. The inter-row mulches reduced volunteer canola seed production by about one third (by 7200 to 9000 seeds m^−2^) compared to the absence of inter-row mulches (Figure 3). This result corresponded with the observed reduction in canola plant fecundity (by 170 to 180 seeds plant^−1^) due to the presence of inter-row mulches (Table 3), which was expected since the inter-row mulches did not influence canola seedling density or plant survival to maturity (based on Tukey’s HSD α = 0.05) (data not shown). Among environments, volunteer canola seedling density averaged 57 seedlings m^−2^, 76% of which survived to plant maturity (data not shown). Volunteer canola survivorship was unaffected by the living mulch treatments. Even though both living and terminated inter-row mulches reduced volunteer canola seed production (Table 3; Figure 3), volunteer canola biomass only responded to the living mulches (Figure 4). The living mulches resulted in about half the volunteer canola biomass compared to the respective mulch-free controls (volunteer canola biomass reduced by 54% and 50% for the living wheat and rye mulches, respectively) (Figure 4). Terminating mulches mid-season lessened these impacts and allowed the volunteer canola to recover and produce biomass similar to the absence of inter-row mulches (Figure 4), while seed production remained impaired (Table 3; Figure 3). These results indicate that some morphological features of volunteer canola (such as vegetative growth and development) were able to recover following mid-season mulch termination while aspects of reproductive fitness were unable to recover following early season interference from the living mulches.

Clodinafop applied post-emergence indirectly reduced volunteer canola seed production and return to the soil seedbank compared to post-emergence glyphosate. Volunteer canola plant fecundity and seed production per unit area were reduced by 51% and 46%, respectively, among mulch types, spatial arrangements, and environments when treated with clodinafop compared to glyphosate (Table 3; Figure 3). The percentage of volunteer canola seeds returned to the soil seedbank in the glyphosate regime (75%) was about double that of the clodinafop regime (36%) in Carman 2014 only (Table S3). Clodinafop has selective activity on grass but not broadleaf species. Post-emergence clodinafop allowed the inter-row mulches to remain live while managing the grass weeds that were present. In contrast, the non-selective activity of glyphosate terminated the living mulches and all weeds other than the glyphosate-resistant volunteer canola. The absence of interference from the living mulches or other broadleaf weeds in the glyphosate regime that were otherwise present in the clodinafop regime facilitated greater niche capture and seed production by the volunteer canola. However, few broadleaf weeds other than volunteer canola were observed in the clodinafop herbicide regime in the current study.

### 3.3. Inter-Row Mulches

**Figure 5:**
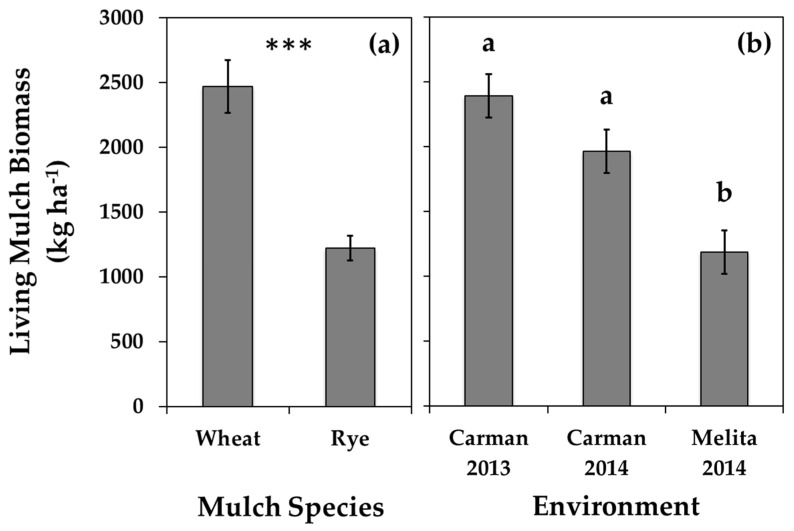
Spring-seeded inter-row living mulch aboveground biomass under volunteer canola interference in soybean for (**a**) each mulch species in a combined environment analysis and (**b**) each environment individually. Error bars indicate ± SEM. Within sub-figures where multiple comparisons are made, different letters indicate significant differences based on Tukey’s HSD (α = 0.05). Within sub-figures where a single mean comparison is made, *** indicates a significant F-test effect at *p* < 0.001.

**Figure 6:**
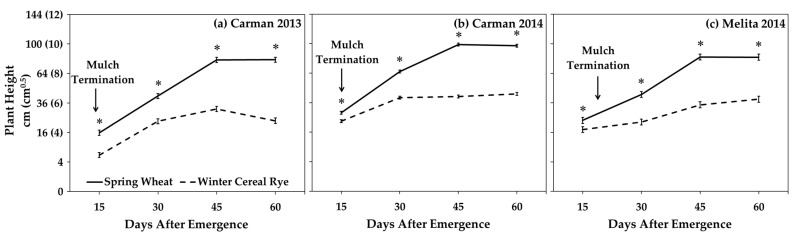
Spring-seeded inter-row living mulch plant heights in soybean under volunteer canola interference. Plant heights were measured at 15, 30, 45, and 60 days after soybean emergence and are shown separately for (**a**) Carman 2013, (**b**) Carman 2014, and (**c**) Melita 2014. The date of mulch termination is indicated for each environment, beyond which the growth of terminated mulches was not observed. *Y*-axis values are back-transformed square root means with the square root-transformed means in parentheses. Error bars indicate ± SEM. An asterisk (*) indicates significant difference between mulch species based on Tukey’s HSD (α = 0.05).

While soybean yield and volunteer canola seed production were similarly affected by both the wheat and cereal rye mulches, these two mulch species likely invoked different interference mechanisms. Double the aboveground biomass (Figure 5) and consistently greater plant density (Table S1) and height (Figure 6) of the spring wheat compared to the winter cereal rye living mulches suggests substantially greater interference potential of the spring wheat mulches. However, all of the soybean and volunteer canola response variables were similarly affected by the wheat and rye mulches (Table 1 and Table 3; Figure 3 and Figure 4), suggesting that compensatory non-resource-limiting (indirect) competition likely contributed to the interference potential of the inter-row rye mulches. Unlike the living rye mulch, the spring wheat mulch entered reproductive development, and when harvested as an intercrop with soybean, it consistently resulted in 720 ± 60 kg ha^−1^ grain yield (data not shown).

Planting date had a greater impact on living mulch biomass than mulch plant densities, which resulted in lower mulch biomass in Melita 2014 compared to the other environments. The Melita 2014 experiment was planted 14 and 16 days later than Carman 2013 and Carman 2014, respectively, based on the calendar year (Figure 1), and this was associated with the lowest amount of living mulch biomass (Figure 5). In contrast, over double the density of the mulches in Carman 2013 did not result in greater living mulch biomass compared to Carman 2014 (Figure 5). The consistent impact of the inter-row mulches on volunteer canola seed production (Table 3; Figure 3) suggests that the benefits of these inter-row mulches for weed management may be realized at both higher and lower mulch densities. However, the only environment where the living mulches reduced soybean yield compared with mid-season mulch termination was also the environment with the greatest mulch plant densities (Carman 2013) (Table S1). Together, these data suggest an opportunity to optimize weed suppression from inter-row living mulches while also mitigating potential mulch-induced soybean yield losses by seeding the inter-row mulch at lower densities.

## 4. Discussion

Our results correspond with previous studies implementing cereal rye living mulch for weed control. The weed suppressive effects of living rye mulch are well documented and are often consistent among environments [15,23,26,27,28,29,30]. However, living mulch-induced cash crop yield loss is dependent on soil moisture, precipitation, and agronomics (e.g., mulch species, density, planting pattern, and mulch suppression or termination method) [15]. Several studies report soil moisture as a key limiting factor governing living mulch-induced yield loss [23,29,30]. The current study corresponds with these reports since the only environment in which the living mulches affected soybean yield was also that which received below normal precipitation throughout the growing season (Table 1; Figure 1). It should be noted, however, that we did not observe mulch-induced soybean yield loss compared to the mulch-free controls, but rather mid-season mulch termination in Carman 2013 resulted in greater yield compared to mulches that remained live throughout the growing season (Table 1). Over double the plant density of the living mulches (Table S1) compounded with low precipitation in Carman 2013 (Figure 1) likely limited the moisture available to the soybean, resulting in reduced yield as the living mulches continued to grow and use available resources throughout the growing season.

Soil moisture is often cited as a driving factor of living mulch-induced cash crop yield loss; however, direct competition for other resources in addition to resource-independent plant interference could also contribute to the impact of living mulch on cash crop yield [15]. Growing soybean in nitrogen-limited environments in western Canada resulted in the greatest reduction in volunteer canola seed production [1]. Canola was among the top three species exhibiting the greatest shoot biomass response to added nitrogen when compared to 23 agricultural weed species [40]. Since NO_3_-N was relatively low among all three environments in the current study (11–37 kg N ha^−1^), it is likely that nitrogen assimilation by the living mulches resulted in less nitrogen being available to the volunteer canola, while the soybean crop met its nitrogen requirements through biological nitrogen fixation. Wells et al. [41] observed a similar interference mechanism for a fall-seeded cereal rye cover crop that was roller-crimped before soybean planting in North Carolina. Alternatively, the allelopathic potential of cereal rye is well documented [20,22,42]. Greater allelopathic activity of rye compared to wheat living mulches would explain the similar impact of these species on soybean and volunteer canola despite the greater plant density, height, and biomass of the wheat living mulches. The exact mechanism of weed interference employed by these living mulches warrants further investigation.

While fall-seeded cover crops terminated around soybean planting in spring have been the focus of several research studies (for example, those summarized elegantly by Mirsky et al. [43]), the current study suggests that spring-seeded living mulches in soybean could also be a viable tool to suppress weeds during soybean establishment in short-season growing environments. In Manitoba, a fall-seeded cereal rye cover crop resulted in the lowest weed biomass at organic dry bean (*Phaseolus vulgaris* L.) harvest compared to fall-seeded barley (*Hordeum vulgare* L.), oat (*Avena sativa* L.), or a no cover crop control [44]. In addition, the rye cover crop also resulted in lower pre-seeding weed density but also about halved dry bean yield compared to the no cover crop control in one of two environments [44]. No-tillage cover crop management (roller-crimped rye) was more effective at reducing weed biomass than cover crop tillage before bean seeding but also resulted in a dry bean yield penalty in one environment [44]. Maximization of fall-seeded cover crop biomass prior to termination in spring is critical to achieve maximum weed suppression in these systems [43], but short-season growing environments in northern climates can limit biomass accumulation by fall-seeded cover crops [45]. Instead, the current study suggests that spring-seeded living mulches could be used achieve the weed suppressive effects of cover crops in northern climates where biomass accumulation of fall-seeded cover crops is variable and often insufficient.

The living mulches were effective at suppressing a competitive early season weed species, volunteer canola, in soybean. The relative differences in the timing of the critical period for weed control (CPWC) in these two species could be one factor driving the reduced seed production of volunteer canola but not soybean in the presence of the living and terminated inter-row mulches [46,47]. The CPWC is the period of crop growth and development during which the crop must be kept weed-free to mitigate unacceptable crop yield loss [48]. The CPWC in canola begins slightly earlier than that for soybean [46,47]. In western Canada, canola yield loss may be mitigated if weeds are removed on or before the 4-leaf stage (around 17 to 38 days after canola emergence) [46]. In soybean, however, the critical timing of weed removal (CTWR) with a 5% yield loss threshold ranged between the third node (V3 stage) to the beginning of seed development (R5 stage) (between 16 and 50 days after soybean emergence) in eastern Canada [47]. Since soybean generally exhibit delayed emergence compared to canola in western Canada [49], living mulches terminated after the CTWR in canola but before the CTWR in soybean could decrease the seed production of volunteer canola absent of soybean yield loss. Since recent reports suggest that cultural management tools such as narrow row spacing can both delay the beginning (CTWR) and hastening the end (critical weed-free period) of the CPWC in soybean [50,51], it is possible that integrating living mulches with other cultural tools could help refine this system by targeting early season weeds while avoiding adverse effects on soybean yield.

Weed communities in the Northern Great Plains region of North America are dominated by cool-season weed species that are competitive in the early spring, while soybean exhibits a prolonged establishment period [1,8,32]. In a 2016 mid-season survey conducted in Manitoba, Canada, volunteer canola was the most abundant weed species present among 118 soybean fields, followed by wild buckwheat (*Fallopia convolvulus* (L.) Á. Löve) and barnyard grass (*Echinochloa crusgalli* (L.) Beauv.) [32]. The peak emergence of all three of these species coincide with recommended soybean planting dates in this region, resulting in rapid growth and development during soybean establishment [52,53]. Volunteer canola was found in 45% of the soybean fields surveyed after post-emergence herbicide application [32]. Similar relative timing of emergence, growth, and development of volunteer canola to that of the most abundant weeds in this region make canola an ideal model weed species that is representative of the dominant cool-season weed community that is present. 

Adoption of integrated weed management is critical to mitigating the proliferation of herbicide-resistant annual weeds, such as volunteer canola; however, the implementation of non-chemical weed control is often limited to those practices that are inexpensive for farmers. In living mulch systems such as those employed in the current study, the cost of seed for living mulches represents the greatest implementation expense. This expense may be lowered by reusing seed from previous cereal crops within the crop rotation rather than by purchasing certified seed for establishing the living mulch. Based on sale prices for spring wheat ($0.23 CAD kg^−1^) and cereal rye ($0.18 CAD kg^−1^) [54], the expense of living mulch establishment at the rates used in the current study ranged from $7.02 CAD (rye mulch in 38 cm spatial arrangement) to $12.25 CAD (wheat mulch in 76 cm spatial arrangement) ha^−1^, which is less than the cost of most post-emergence herbicides. In addition, the living wheat mulch produced enough wheat seed to resupply the establishment of the living mulch in subsequent years or alternatively, to add an additional income source of about $165.60 ha^−1^ CAD along with the soybean grain yield. Thus, the cost of implementing inter-row living mulches in soybean can be relatively inexpensive, making it a worthwhile addition to integrated weed management programs.

## 5. Conclusions

Spring-seeded wheat and cereal rye inter-row living mulches were a viable option to facilitate the early season suppression of volunteer canola in soybean grown in the Northern Great Plains region of North America. When employing this strategy, close monitoring of precipitation is warranted to mitigate potential mulch-induced soybean yield losses, while early season precipitation could be used as a trigger for the termination of living mulches mid-season, if required. While the living and terminated mulches suppressed volunteer canola seed production by about one third (by 7200 to 9000 seeds m^−2^) compared to the respective mulch-free controls, this alone would not be enough to reduce volunteer canola seed return to the soil seedbank to the extent required for seedbank depletion. Therefore, living mulches should be employed with other non-chemical tools in a comprehensive integrated weed management program targeting problematic herbicide-resistant weeds in soybean. Mitigating canola harvest losses [4,6], seedbank depletion with timely soil disturbance [7], planting soybean in nitrogen-limited environments [1], and increased soybean seeding rates [55] also contribute to volunteer canola management in the Northern Great Plains region and represent viable options to be implemented along with living mulches. This study demonstrates the utility of spring-seeded wheat and cereal rye inter-row living mulches for enhanced early season weed suppression in soybean as one component of an integrated weed management program in the Northern Great Plains region.

## Figures and Tables

**Table 1:** Soybean grain yield in the presence of volunteer canola as influenced by soybean/living mulch spatial arrangement and the presence or absence of spring-seeded wheat or rye inter-row mulches (mulch type) with (glyphosate) or without (clodinafop) mid-season mulch termination using two different herbicide regimes in three environments individually and in a combined analysis.

Spatial Arrangement	Herbicide Regime	Mulch Type ^b^	Soybean Yield ^a^ (kg ha^−1^)			
			Carman 2013	Carman 2014	Melita 2014	Combined
38 cm			2080	1010 A	1320	1410
76 cm			1800	650 B	1130	1260
						
	Clodinafop	Unseeded	1920 ab	680	1170	1260 bc
	Clodinafop	Wheat (L)	1490 b	680	1160	1110 c
	Clodinafop	Rye (L)	1480 b	580	1050	1040 c
	Glyphosate	Unseeded	2140 a	920	1320	1460 ab
	Glyphosate	Wheat (T)	2330 a	1120	1510	1650 a
	Glyphosate	Rye (T)	2270 a	990	1120	1460 ab

^a^ Within columns and effect groupings, different letters indicate significant differences based on Tukey’s HSD (α = 0.05). ^b^ Abbreviations: L, living; T, terminated mid-season.

**Table 2:** Soybean aboveground biomass in the presence of volunteer canola as influenced by soybean/living mulch spatial arrangement or two different herbicide regimes in three environments individually and in a combined analysis.

Spatial Arrangement	Herbicide Regime	Soybean Biomass ^a^ (kg ha^−1^)			
		Carman 2013	Carman 2014	Melita 2014	Combined
38 cm		- ^b^	-	-	3080 A
76 cm		-	-	-	2530 B
					
	Clodinafop	3490 b	1200	2470	2380 b
	Glyphosate	5450 a	1690	2520	3220 a

^a^ Within columns and effect groupings, different letters indicate significant differences based on Tukey’s HSD (α = 0.05). ^b^ A dash (-) indicates lack of significant F-test effect (*p* ≥ 0.05).

**Table 3:** Volunteer canola plant fecundity in soybean as influenced by the presence or absence of spring-seeded wheat or rye inter-row mulches (mulch type) or to two different herbicide regimes in three environments individually and in a combined analysis.

Herbicide Regime	Mulch Type	Volunteer Canola Plant Fecundity ^a^ (Seeds Plant^−1^)			
		Carman 2013	Carman 2014	Melita 2014	Combined
Clodinafop		270 B	330 B	550	380 B
Glyphosate		590 A	870 A	890	780 A
					
	Unseeded	- ^b^	-	-	700 a
	Wheat	-	-	-	530 b
	Rye	-	-	-	520 b

^a^ Within columns and effect groupings, different letters indicate significant differences based on Tukey’s HSD (α = 0.05). ^b^ A dash (-) indicates lack of significant F-test effect (*p* ≥ 0.05).

## Data Availability

The data presented in this study are available upon request from the corresponding author.

## Supplementary Materials

The following are available online at https://www.mdpi.com/article/10.3390/plants10112276/s1, Table S1: Spring-seeded wheat or rye inter-row living mulch seedling density in soybean under volunteer canola interference as influenced by soybean/living mulch spatial arrangement and mulch species in each environment individually and in a combined analysis; Table S2: Soybean seedling density under volunteer canola interference as influenced by soybean/living mulch spatial arrangement, herbicide regime, and the presence or absence of spring-seeded wheat or rye inter-row mulches in each environment individually; Table S3: The percentage of volunteer canola seeds that were returned to the soil seedbank due to seed losses prior to or during soybean harvest in two different herbicide regimes in three environments individually and in a combined analysis.
