# Supplementary material for: Wheat and Cereal Rye Inter-Row Living Mulches Interfere with Early Season Weeds in Soybean
Source: Plants (Basel). 2021 Oct 24;10(11):2276. doi: 10.3390/plants10112276 (PMC8620416; doi:10.3390/plants10112276)
Supplement: Supplementary file 1 [file plants-10-02276-s001.zip › plants-1404633-supplementary.pdf]

Table S1. Spring-seeded wheat or rye inter-row living mulch seedling density in soybean under volunteer canola interference as influenced by soybean/living mulch spatial arrangement, and mulch species in each environment individually and in a combined analysis.

Spatial Arrangement	Mulch Type	Mulch Plant Density [a] (Plants m^{-2})			
		Carman 2013	Carman 2014	Melita 2014	Combined
38 cm		115	69 A	66 A	- [b]
76 cm		158	35 B	39 B	-
	Wheat	-	-	-	91 a
	Rye	-	-	-	70 b

[a] Within columns and effect groupings, different letters indicate significant differences based on Tukey's HSD (α = 0.05). [b] A dash (-) indicates lack of significant F-test effect.

Table S2. Soybean seedling density under volunteer canola interference as influenced by soybean/living mulch spatial arrangement, herbicide regime, and the presence or absence of spring-seeded wheat or rye inter-row mulches in each environment individually.

Spatial Arrangement	Herbicide Regime	Mulch Type [a]	Soybean Plant Density [b] (Plants m^{-2})		
			Carman 2013	Carman 2014	Melita 2014
38 cm	Clodinafop	Absent	43	48 a	16
		Wheat (L)	48	42 a	18
		Rye (L)	42	31 bc	13
	Glyphosate	Absent	43	33 ab	15
		Wheat (T)	48	31 abcd	16
		Rye (T)	42	40 ab	15
76 cm	Clodinafop	Absent	45	18 cd	19
		Wheat (L)	45	14 d	13
		Rye (L)	39	22 cd	16
	Glyphosate	Absent	45	19 cd	17
		Wheat (T)	45	22 cd	13
		Rye (T)	39	27 cd	16

[a] Abbreviations: L, living; T, terminated mid-season. [b] Within columns, different letters indicate significant differences based on Tukey's HSD (α = 0.05).

Table S3. The percentage of volunteer canola seeds that were returned to the soil seedbank due to seed losses prior to or during soybean harvest in two different herbicide regimes in three environments individually and in a combined analysis.

Herbicide Regime	Volunteer Canola Seed Return [a] (%)			
	**Carman 2013**	**Carman 2014**	**Melita 2014**	**Combined**
Clodinafop	59	36 b	59	51 b
Glyphosate	58	75 a	67	67 a

[a] Within columns, different letters indicate significant differences based on Tukey's HSD (α = 0.05).
